# Impact of the 3D Microenvironment on Phenotype, Gene Expression, and EGFR Inhibition of Colorectal Cancer Cell Lines

**DOI:** 10.1371/journal.pone.0059689

**Published:** 2013-03-26

**Authors:** Anna C. Luca, Sabrina Mersch, René Deenen, Stephan Schmidt, Isabelle Messner, Karl-Ludwig Schäfer, Stephan E. Baldus, Wolfgang Huckenbeck, Roland P. Piekorz, Wolfram T. Knoefel, Andreas Krieg, Nikolas H. Stoecklein

**Affiliations:** 1 Department of Surgery A, Heinrich-Heine University and University Hospital Düsseldorf, Düsseldorf, Germany; 2 Biological and Medical Research Center (BMFZ), Heinrich-Heine University Düsseldorf, Düsseldorf, Germany; 3 Institute of Biochemistry and Molecular Biology II, Heinrich-Heine University and University Hospital Düsseldorf, Düsseldorf, Germany; 4 Institute of Pathology, Heinrich-Heine University and University Hospital Düsseldorf, Düsseldorf, Germany; 5 Institute of Forensic Medicine, Heinrich-Heine University and University Hospital Düsseldorf, Düsseldorf, Germany; Dresden University of Technology, Germany

## Abstract

Three-dimensional (3D) tumor cell cultures grown in laminin-rich-extracellular matrix (lrECM) are considered to reflect human tumors more realistic as compared to cells grown as monolayer on plastic. Here, we systematically investigated the impact of ECM on phenotype, gene expression, EGFR signaling pathway, and on EGFR inhibition in commonly used colorectal cancer (CRC) cell lines. LrECM on-top (3D) culture assays were performed with the CRC cell lines SW-480, HT-29, DLD-1, LOVO, CACO-2, COLO-205 and COLO-206F. Morphology of lrECM cultivated CRC cell lines was determined by phase contrast and confocal laser scanning fluorescence microscopy. Proliferation of cells was examined by MTT assay, invasive capacity of the cell lines was assayed using Matrigel-coated Boyden chambers, and migratory activity was determined employing the Fence assay. Differential gene expression was analyzed at the transcriptional level by the Agilent array platform. EGFR was inhibited by using the specific small molecule inhibitor AG1478. A specific spheroid growth pattern was observed for all investigated CRC cell lines. DLD-1, HT-29 and SW-480 and CACO-2 exhibited a clear solid tumor cell formation, while LOVO, COLO-205 and COLO-206F were characterized by forming grape-like structures. Although the occurrence of a spheroid morphology did not correlate with an altered migratory, invasive, or proliferative capacity of CRC cell lines, gene expression was clearly altered in cells grown on lrECM as compared to 2D cultures. Interestingly, in KRAS wild-type cell lines, inhibition of EGFR was less effective in lrECM (3D) cultures as compared to 2D cell cultures. Thus, comparing both 2D and 3D cell culture models, our data support the influence of the ECM on cancer growth. Compared to conventional 2D cell culture, the lrECM (3D) cell culture model offers the opportunity to investigate permanent CRC cell lines under more physiological conditions, i.e. in the context of molecular therapeutic targets and their pharmacological inhibition.

## Introduction

Permanent cancer cell lines can provide an almost unlimited supply of cells with quite similar genotypes and phenotypes [1] and are essentially the only option for mechanistic studies of human cancer cells under controlled conditions [2], [3]. Consequently, human cancer cell lines have been widely used as *in vitro* models for human cancer. An example for a successful and well-noted area of application has been the discovery, development and testing of cancer drugs [3]. Nonetheless, depending on the scientific question there are also obvious limitations in studying changes on cancer cells that are associated with cancer progression since most permanent cancer cell lines have been established from advanced cancers with progressed genotypes [1]. However, one of the most important problems restricting the value of cancer cell lines as a model for human cancer is due to the most common method to culture cell lines *in vitro*, i.e. as homotypic monolayer on plastic substrates (2D). Whereas primary cancers and metastases are complex heterotypic three-dimensional structures embedded in distinct organ-specific microenvironments, it is well known that cells that are grown as classical 2D cultures loose many of the hallmarks of their *in vivo* counterparts [4]. In addition, important cellular functions such as proliferation and differentiation can be artificially altered [5].

A common feature of all normal and malignant epithelial cells is that they are physiologically in close contact to the extracellular matrix (ECM). The ECM, composed of fibrous proteins and glycosaminoglycans, surrounds epithelial cells in their extracellular space and forms their basal membrane. The ECM provides not only physical strength to organized epithelial cells [6], [7], but also important key biochemical structures and signals for polarity and growth [7], [8]. A simple system for *in vitro* ECM modelling is a solubilised basement membrane preparation extracted from the Engelbreth-Holm-Swarm (EHS) mouse sarcoma, a tumor rich in extracellular matrix proteins comprising laminin, collagen IV, heparin sulphate proteoglycans and entactin/nidogen [9]–[18]. Because of its molecular composition, especially its high laminin content, it is considered to be a suitable substitute for the basement membrane. If epithelial cells are cultured within this laminin-rich extracellular matrix (lrECM), they grow as three-dimensional structures [15], [16], [19]. Pioneering work by the Bissell group and others – mainly done on primary breast cells and breast cancer cell lines – demonstrated dramatic morphological and biochemical differences, between normal and malignant cells grown 2D on plastic substrates and 3D in lrECM, respectively [6], [20], [21]. From a clinical point of view it is important to note that lrECM (3D) culture – as a model closer to the *in vivo* situation – can lead to different responses to molecular therapies, as recently shown for breast cancer cell lines [22], [23], [24]. Surprisingly, lrECM (3D) cultures are still rarely used in experiments with cancer cell lines and only few studies systematically analyzed the effects of lrECM cultures on permanent cell lines providing basic information on these models. So far, such systematic analyses of lrECM cultures focused mainly on the phenotypic characterization of breast cancer cell lines grown under the lrECM 3D *vs.* 2D conditions.

Here, we expanded the functional understanding of the effects of differential lrECM (3D) *vs.* 2D growth conditions to colon cancer cells. We systematically investigated the impact of lrECM on cell phenotype and gene expression patterns in commonly used colorectal cancer (CRC) cell lines. Our data indicate that CRC cell lines exhibit distinct morphologic spheroid types when cultured in lrECM. Although spheroid morphology of CRC lines did not correlate with an altered migratory, invasive or proliferative cell capacity, cell lines grown under lrECM (3D) conditions exhibited *per se* an impaired proliferation when compared to control 2D cultures. Moreover, gene expression was clearly altered in CRC cell lines when cultivated under lrECM/3D conditions. In addition, the efficacy of pharmacological EGFR inhibition was impaired in CRC cells grown on lrECM when compared to 2D cultures. Thus, the 3D microenvironment has a major impact on cellular phenotype and pharmacological sensitivity of CRC cell lines.

## Materials and Methods

### Cell Lines and Cell Culture

LOVO was obtained from the European Collection of Cell Cultures (ECACC, Salisbury, UK), COLO-205 from the American Type Culture Collection (ATCC, LGC Standards GmbH, Wesel, Germany), CACO-2, COLO-206F, DLD-1, HT-29 and SW-480 from the German Resource Centre for Biological Material (DSMZ, Braunschweig, Germany). All cell lines were maintained under standard tissue-culture conditions in RPMI 1640+ GlutaMAX™-I (Gibco/Invitrogen, Darmstadt, Germany) containing 10% fetal calf serum (Gibco/Invitrogen,). Cells were cultivated either on tissue culture plastic (2D) (Greiner bio-one, Frickenhausen, Germany) or 3D within growth factor reduced laminin-rich extracellular matrix (lrECM 3D) on-top cultures by seeding cells on top of a thin gel of Engelbreth-Holm-Swarm tumor extract (BioCoat Matrigel Basement Membrane, growth factor reduced, BD Biosciences, Heidelberg, Germany). Cells were plated in the Matrigel on-top assay at a density of 1.8×10^4^ cells/well in 24 well plates. Spheres were cultured for 7 days before recovering from Matrigel. For morphology studies, spheres were cultured up to 10 days. Medium was changed every other day in 3D cultures. 3D-spheres were recovered from the Matrigel Basement Membrane by removing the medium from the Matrigel cell culture and incubation in 400 µl/well dispase (BD Biosciences, Heidelberg, Germany), preheated at 37°C, for 2 h at 37°C and 5% CO_2_. The activation of the dispase was stopped with 10 mM EDTA in 1×PBS. Spheres were centrifuged and washed with 1×PBS. 2D cultured cells were also washed with PBS and scraped of the culture dish. For protein isolation 3D spheres were recovered from Matrigel by incubating with 5 mM EDTA in PBS for 30 min on ice followed by three washing steps with PBS.

### KRAS and BRAF Mutation Analysis

Standard cycle sequencing was performed to (re-)evaluate the *KRAS* and *BRAF* mutation status of the colon carcinoma cell lines **(** **Table 1** **)**. Exons 2, 3 and 4 of *KRAS* were analyzed using the following oligonucleotide primers: 5′- AGGCCTGCTGAAAATGACTGAA-3′ and 5′- AAAGAATGGTCCTGCACCAG-3′ for Exon 2, 5′-GGATTCCTACAGGAAGCAAGT-3′ and 5′-GGCAAATACACAAAGAAAGC-3′ for Exon 3 and 5′- AGACACAAAACAGGCTCAGGA-3′ and 5′- AAGAAGCAATGCCCTCTCAA-3′ for Exon 4. For *BRAF* the amplification of Exon 15 was performed by using forward primer 5′- TGCTTGCTCTGATAGGAAAATG-3′ and reverse primer 5′- AGCCTCAATTCTTACCATCCA-3′. Whereas reverse primers were used for DNA sequence analysis of *KRAS* Exon 2 and 4, forward primers were used for *KRAS* Exon 3 and BRAF Exon 15, respectively [25].

**Table 1:** Clinical and molecular characterization of the CRC cell lines.

	CACO-2	DLD-1	HT-29	SW-480	LOVO	COLO-205	COLO-206F
Origin of CRC cell line	PT	PT	PT	PT	M (nodule)	M (ascites)	M (ascites)
Gender	male	male	female	male	male	male	male
Age at diagnosis	72	adult	44	50	56	70	70
KRAS	WT	Gly13Asp	WT	Gly12Val	Gly13Asp	WT	WT
BRAF	WT	WT	Val600Glu	WT	WT	Val600Glu	Val600Glu
EGFR	WT	WT	WT	WT	WT	WT	WT

### STR Analysis

For STR fingerprinting analysis DNA was isolated using the Qiagen Blood and Tissue Kit (Qiagen, Hilden, Germany) according to the manufactures instruction. Genomic DNA (1 ng) was amplified using the genRES® MPX-2 and genRES® MPX-3 multiplex PCR systems (Serac GmbH, Bad Homburg, Germany) to generate 9 and 12 different STR marker sequences. PCR products were analyzed on an ABI 310 capillary sequencer and allelotyped by the “genotyper V3.1″ software (Applied Biosystems, Carlsbad, CA, USA) [26].

### Cell Viability, Proliferation and Apoptosis Assays

Cell viability was measured using the MTT assay. Cells were plated at a density of 2×10^3^ cells/well in 96 well plates. Whereas cells were plated directly on tissue plastic plates for 2D cultures, 3D cultures were prepared by plating the cells on a thin layer of Matrigel. After an incubation period for 48 h at 37°C and 5% CO_2_, 3-(4,5-Dimethylthiazol-2-yl)-2,5-diphenyltetrazolium bromide (MTT) (Sigma-Aldrich, Hamburg, Germany) was added to the media for 4 h before fixation overnight with MTT stopping solution containing 10% SDS, 5% 1-butanol and 0.01 M HCl. Reduction of MTT to formazan was measured at 540 nm using a Tecan sunrise remote reader (Tecan group Ltd., Maennedorf, Austria).

Proliferation of cells grown in 2D or 3D culture conditions was quantified by 5-Bromo-2-deoxyuracil (BrdU) incorporation using the 5-Bromo-20-deoxy-uridine Labeling and Detection Kit I (Roche) following the manufacturers’ instructions. After an incubation period for 48 h at 37°C and 5% CO_2_ media was removed and cells were incubated for additional 1 h at 37°C and 5% CO_2_ with BrdU labeling media. 3D spheres were recovered from Matrigel by incubating with 5 mM EDTA in PBS for 30 min. Cells were washed two times in 1×PBS and centrifuged for 5 min at 400 rpm. Recovered spheroids were coated on glass slides and dried overnight at room temperature. Nuclei were counterstained with 40,6-diamidino-2-phenylindole (DAPI). Up to 200 cells were counted in five different fields per preparation using an Axio Scope fluorescence microscope (Zeiss, Jena, Germany). The number of proliferating cells was calculated as a percentage of the total number of DAPI-positive cells per field (three randomly selected fields per coverslip). The percentage of apoptotic cells was calculated as the ratio between the number of DAPI-positive cells with fragmented nuclei, known as a morphological hallmark of apoptosis, and all DAPI-positive cells.

### Fence Assay (Migration Assay)

For migration assays 7.5×10^3^ cells were plated into a 16 mm fence (Aix Scientifics®, Aachen Germany). Cells were cultivated in RPMI media supplemented with 10% FBS until confluence was reached and the fence was removed. Cells were grown for four days, subsequently fixed in 10% formaldehyde and stained with Mayer’s haemalaun staining solution. Diameters of stained cell monolayers were assessed in mm using the Versa Doc Imaging System (BioRad, Munich, Germany). To distinguish between cell proliferation and cell migration, pre-experiments were performed in culture media supplemented with 1% FBS or 10% FBS.

### Invasion Assay

Invasion assay was performed using BD BioCoat™ Matrigel™ Invasion Chamber (BD Biosciences) following the instructions of the manufacturer. Membranes were fixed on glass slides and stained with crystal violet. Cells within a defined central area of the membrane were counted.

### Drug Treatment and Proliferation Assay

EGF receptor was inhibited by tyrosine kinase inhibitor tyrphostin AG1478 (Sigma Aldrich). Cells were plated at a density of 6×10^3^ cells/well in 96 well plates and treated with AG1478 at a final concentration of 20 µM, 10 µM, 5 µM, 2,5 µM, 1 µM or 0,1 µM for 48 h and 96 h in 2D and 3D cell culture systems. DMSO/methanol at equimolar concentrations to AG1478 concentrations served as vehicle control. Forty-eight hours after drug treatment cell proliferation was measured by performing MTT assays as described above.

### Total RNA Extraction and cDNA Synthesis

Total RNA was isolated using RNeasy Mini Kit (Qiagen) according to manufacture’s instructions. RNA was diluted in 50–80 µl nuclease free and sterile water. RNA concentration was measured spectrophometrically at 260 nm and RNA quality was assessed using the Agilent 2100 Bioanalyzer. For cDNA synthesis, reverse transcription (RT) was performed in a final volume of 20 µl by using 1∶10 diluted oligo(dT) primer (Invitrogen), 2 µg RNA, and 4 U transcriptor reverse transcriptase in 5×RT buffer (Roche, Mannheim, Germany).

### Real Time PCR

Primers and according probes were designed using Universal ProbeLibrary Assay Design Center (ProbeFinder version 2.45, Roche Applied Science). Primers were all synthesized by MWG-Biotech, Ebersberg, Germany **(Table S1)**. cDNA was diluted to a final concentration of 1 ng/µl. For PCR, 2 µl cDNA template (or water as a negative control) was mixed with 12.5 µl Fast Start Taq Man Probe Master mix (Roche), 0.25 µl of each forward and reverse primer (20 µM each) and 0.25 µl probe (Universal ProbeLibrary Set human, Roche). All samples were run in triplicates. To normalize the expression of target genes, we used glyceraldehyde-3-phosphate dehydrogenase (GAPDH) as the internal reference gene. Quantitative real-time-PCR (qPCR) was performed on the DyadDisciple Chromo 4 (BioRad) using the following conditions: 95°C for 10 min followed by 40 cycles each comprising denaturation for 15 sec at 95°C, annealing and extension for 1 min at 60°C. Gene expression was quantified by using the 2^−ΔΔCT^ method [27].

### Immunoblotting

Cells were homogenized in protein lysis buffer Pb1 (100 mM NaCl, 10 mM Tris, 1 mM EDTA, 1% Nonident P40 (Sigma) supplemented with one tablet of Phosphatase Inhibitor Cocktail Tablets and PhosSTOP Phosphatase Inhibitor Cocktail (both Roche). Soluble proteins were separated by centrifugation for 10 min at 4°C and 12000 rpm. Samples containing 40 µg of clarified protein lysates per lane were separated electrophoretically on SDS-PAGE gels and transferred to nitrocellulose membranes. Membranes were probed with monoclonal rabbit anti-EGF receptor antibody (Clone C74B9; Cell Signaling, Denver, MA, USA) overnight at 4°C. For detection of AKT1 monoclonal rabbit anti-phospho AKT1 (Ser473) antibody (Clone 193H12; Cell Signaling) and monoclonal rabbit anti-AKT1 antibody (Clone C73H10; Cell Signaling) were used. MAPK p44/42 was detected using monoclonal rabbit anti-phospho p44/42 MAPK (Thr202/Tyr204) antibody (Clone 20G11; Cell Signaling) and monoclonal rabbit anti-p44/42 MAPK antibody (Clone 137F5; Cell Signaling). Detection of MEK1/2 was performed by using polyclonal rabbit anti-phospho MEK1/2 (Ser217/221) antibody (Cell Signaling) and monoclonal rabbit anti-MEK1/2 antibody (Clone 47E6; Cell Signaling). For non-phospho (active) β-Catenin (Ser33/37/Thr41) we used monoclonal rabbit antibody clone D13A1 (Cell Signaling) and for total β-Catenin monoclonal rabbit antibody clone D10A8 (Cell Signaling). Beta-actin was detected using monoclonal mouse anti-β-actin antibody (Clone AC-15; Sigma). Secondary anti-rabbit antibody (Cell Signaling) or anti-mouse antibody (Sigma) linked to horseradish peroxidase were incubated for 90 min at room temperature and detected by using the Immune-Star™ Western C™ Kit (BioRad) using Versa Doc Imaging System (BioRad).

### Confocal Immunofluorescence Microscopy

Recovered spheroids were coated on glass slides, dried overnight at room temperature and stored at −20°C until use. Slides were rehydrated in 1×PBS and fixed with ice-cold methanol/acetone (1∶1) for 20 min at −20°C followed by two wash steps in 1×PBS each for 5 min. Blocking was performed in immunofluorescence buffer containing 4% bovine serum albumin and 0.05% saponin in PBS for 20 min at room temperature. Staining was performed in PBS containing 1% BSA and 0.05% saponin overnight at 4°C. Mouse monoclonal anti-EpCAM antibody BerEp4 (Dako, Hamburg, Germany) was used at a concentration of 2 µg/ml. MOPC (Sigma) served as isotype control and was incubated at a concentration of 2 µg/ml. Next, samples were washed three times with 1×PBS. Secondary antibody, Alexa Fluor® 488 goat anti-mouse IgG (Invitrogen), was incubated for 1 h at room temperature at a concentration of 10 µg/ml, followed by two wash steps each for 5 min. Cells were counterstained with 0.1 µg/ml DAPI (Sigma) in 1×PBS for 4 min, washed two times with PBS, and mounted with VECTASHIELD® solution (Vector Laboratories, Burlingame, CA, USA). Images were obtained using a LSM510-Meta confocal microscope (Zeiss, Jena, Germany) equipped with a 40×/1.3 immersion objective and excitation wavelengths of 364 nm and 488 nm. Confocal pictures shown are single optical slides of 0.5 µm thickness.

### Classification of Spheroids

In a previous work by the Bissell group [2] it was shown that permanent breast cancer cell lines that were cultured in the standardized lrECM on-top assay, developed by this group, formed spheroids, which exhibited characteristic growth-patterns as revealed by phase contrast microscopy or confocal laser scanning microscopy. The spheroid morphology was classified in four distinct discernable groups as described by Kenny *et al.* [2]: ‘Round’, ‘mass’, ‘grape-like’ and ‘stellate’. The ‘round’ type forms colonies on top of the gels characterized by nuclei that are regularly organized around the center of the colony. The ‘mass’ type cell lines form colonies that may also have round colony outlines upon phase contrast microscopy, but have filled centers and disorganized nuclei. The ‘grape-like’ spheroids displayed loose cell-cell contacts with a typical grape-like morphology. ‘Stellate’ spheroids were characterized by an invasive phenotype with stellate projections invading the gel [2]. We used this system to classify the CRC cell line spheroids.

### Gene Expression Analyses: Agilent Array Analyses

Total RNA preparations were analyzed for RNA integrity by Agilent 2100 Bioanalyzer. All samples in this study exhibited common high quality RNA Integrity Numbers (RINs) of 10. RNA was quantified by photometric Nanodrop measurement. Synthesis of cDNA and subsequent fluorescent labeling of cRNA was performed according to the manufacturer’s protocol (One-Color Microarray-Based Gene Expression Analysis, Low Input Quick Amp Labeling, Vers. 6.5; Agilent Technologies). Briefly, 100 ng of total RNA were converted to cDNA, followed by *in vitro* transcription and incorporation of Cy3 labeled nucleotides into newly synthesized cRNA. After fragmentation, labeled cRNA was hybridized to Agilent Human 8×60 K High Density Oligonucleotide Microarrays. Data analyses were conducted with GeneSpring GX software (Vers. 10.5; Agilent Technologies). Signal intensity distributions across samples were normalized by quantile normalization. Input data pre-processing was concluded by baseline transformation to the median of all samples. After grouping of samples according to culture condition (lrECM 3D *vs.* 2D culture, 24 *vs.* 25 samples, respectively) a given transcript had to be expressed in at least 75% of samples in any one of two or both groups to be further analyzed. Detectable gene expression above background was thereby characterized by ‘Present’ flags according to GeneSpring standard settings for Agilent microarrays. Differential gene expression was statistically determined by Mann-Whitney-U-test. Resulting *P*-values were corrected for multiple testing according to Benjamini-Hochberg. Transcripts showing a corrected *P*_corr_-value less than 0.05 were regarded as being differentially expressed. Hierarchical cluster analyses were either performed on samples or on transcripts, respectively, using Manhattan distance metrics and complete linkage.

### Statistics

All experiments were done independently in triplicates unless otherwise indicated. Data represent means ± SD unless otherwise specified. Correlation between spheroid morphology and cell line source (primary tumor *vs.* metastasis) was determined by using the Fisher’s exact test (prism 5, Graph pad Software, Inc. La Jolla, CA, USA). Differences in cell viability measured by dye absorbance at 540 nm, cell proliferation, apoptosis and in gene expression measured by RT-PCR were analyzed using either a paired t-test or the nonparametric two-tailed Mann-Whitney-U-test. A *P*-value less than 0.05 was considered to indicate statistical significance. IC_50_ values were calculated with Prism 5 (Graph pad Software, Inc.).

## Results

### CRC Cell Lines Exhibit Two Distinct Morphologic Spheroid Types when Cultured in lrECM

**Figure 1:**
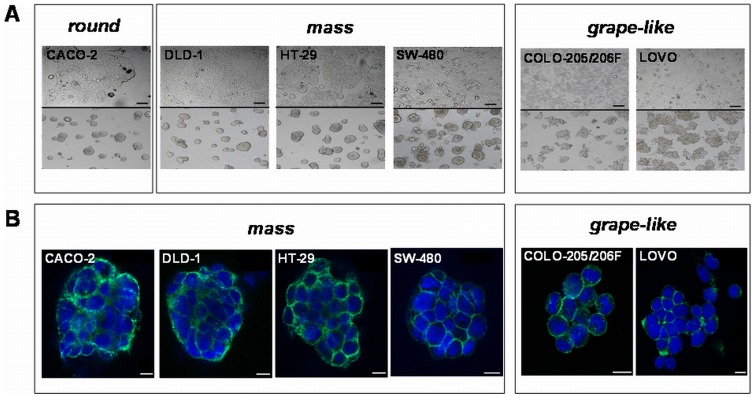
Morphology of 2D and lrECM 3D cultivated CRC cells. **A)** Growth morphology of CRC cell lines cultivated under 2D (upper panel) and lrECM 3D on-top assay conditions (lower panel). Cells cultivated in 3D condition either show a round (CACO-2), mass (DLD-1, HT-29, SW-480) or a grape-like morphology (COLO 205, COLO-206F, LOVO) in phase contrast images. Scale bars: 100 µm. **B)** Confocal laser scanning fluorescence microscopy images of CRC spheroids. Spheroids were grown in lrECM 3D microenvironments for seven days. Subsequent to isolation, the membranous EpCAM protein (green) was stained using Alexa Fluor® 488 goat anti-mouse IgG. Nuclei were counterstained with DAPI (blue). Scale bars 10 µm.

First, we tested whether the CRC cell lines growing on lrECM display a specific morphology which can be classified according to the four categories described by Kenny *et al* [2]. Using the on-top assay all investigated CRC cell lines formed tumor spheroids within two days. Within three more days the spheroids developed a specific morphology, which was replicable in at least ten independent experiments. While spheroids were slowly growing in size, this morphology was stable up to 10 days of cultivation. Longer observation experiments were not done. Among these cell lines, three different growth patterns were observed by phase-contrast microscopy **(** **Figure 1A** **)**: “Round”, “mass” and “grape-like”. In particular, CACO-2 was categorized as “round”, HT-29, DLD-1, and SW-480 as “mass” and LOVO, COLO-206F and COLO-205 as “grape like”.

To increase the spatial resolution and morphological appearance of the different 3D spheroids, we performed confocal microscopy of cells by localization of the epithelial cell adhesion molecule EpCAM and nucleic staining with DAPI **(** **Figure 1B** **)**. Accordingly, CACO-2 spheroids clearly displayed disorganized nuclei and an overall compact structure with colony centers appearing densely filled, being therefore re-classified as “mass”. In all other cell lines confocal imaging validated their initial classification by phase contrast microscopy. Interestingly, we observed that cell lines with quite different 2D morphologies (i.e. COLO-205 and LOVO) exhibited similar morphologies in lrECM and *vice versa*.

### Spheroid Morphology does not Correlate with Migratory, Invasive, or Proliferative Capacity of CRC Cell Lines

After we defined the two divergent spheroid morphologies of lrECM cultured CRC cell lines, i.e. “mass” or “grape-like”, we were interested if this observation was related to different cellular properties in terms of an altered malignant potential. In this context it is noteworthy that all investigated CRC cell lines with “grape-like” morphology derived from metastatic cells, whereas all CRC cell lines with “mass” spheroid morphology were established from primary tumor tissue (Fishers exact test, p = 0.028) **(** **Table 1** **)**.

**Figure 2:**
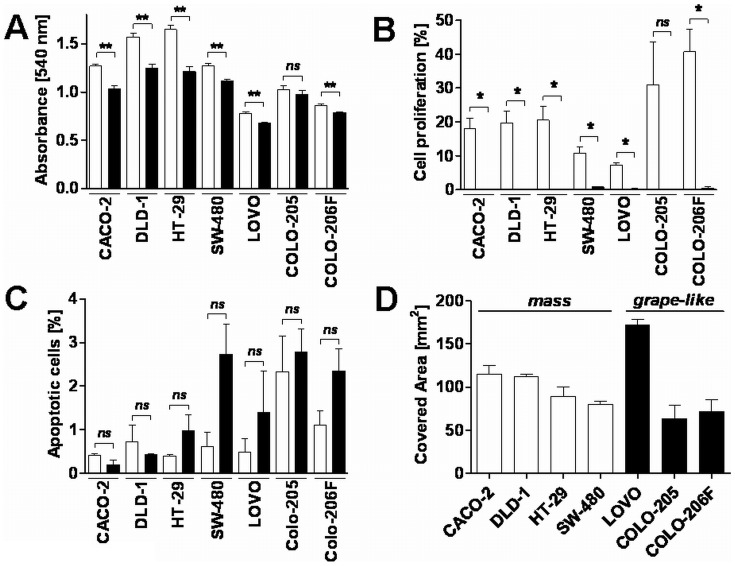
The influence of lrECM microenvironment on cell proliferation, apoptosis and cell migration in CRC cell lines. Cells were cultivated under 2D or 3D conditions. **A)** Cell viability was measured 48 h later using the MTT assay. Values represent the mean absorbance at 540 nm ± SD of triplicates. White bars represent cells cultured on plastic (2D); black bars cells cultured on lrECM (3D). Two-tailed *P*-values were calculated by the Mann-Whitney-U test (** indicates a *P*-value <0.001; ns = not significant). **B)** Cell proliferation was quantified by calculation of the percentage of cells with BrdU incorporation and the total number of DAPI-positive cells per field (at least three randomly selected fields per coverslip). The data presented are the mean ± SD from three replicates. White bars represent cells cultured on plastic (2D); black bars cells cultured on lrECM (3D). Two-tailed *P*-values were calculated by the paired t-test (* indicates a *P*-value <0.05; ns = not significant). **C)** The percentage of apoptotic cells was calculated as the ratio of DAPI-positive cells with fragmented nuclei and all DAPI-positive cells. The data presented are the mean ± SD from three replicates.White bars represent cells cultured on plastic (2D); black bars cells cultured on lrECM (3D). Two-tailed *P*-values were calculated by the paired t-test (ns = not significant). **D)** Migration of CRC cell lines was quantified using the fence assay. Data represent means ± SD of three independent experiments. White bars represent cell lines classified as mass type; black bars the grape-like class.

First, cell viability was determined using the MTT assay. No significant differences in the cell viability between cells of the “mass” phenotype compared to cells of the “grape-like” phenotype became obvious. Interestingly, when comparing viability, proliferation and apoptosis of cells growing two-dimensionally on tissue culture plastic (2D) with the lrECM 3D on-top assay, we found a significant decrease in cell viability and proliferation under lrECM 3D conditions **(** **Figure 2A and B** **)**. In contrast, no difference became obvious when quantifying apoptotic cells in 2D and lrECM 3D culture systems **(** **Figure 2** **C)**.

According to the morphological types observed in lrECM 3D cultures, we explored the migratory and invasive capacity of the CRC cell lines. Using a fence assay to explore migration and a Boyden chamber assay coated with Matrigel to quantify invasion, these experiments did not reveal any obvious differences in terms of the migratory or invasive capacity when comparing the “mass” and “grape-like” CRC cell lines **(** **Figure 2D** **and** **Table 2** **)**. Thus, a correlation between invasiveness and the morphologic type became not evident.

**Table 2:** Invasive capacity of CRC cell lines.

Cell line	Invasion
CACO-2	+
HT-29	++++++
DLD-1	++
SW-480	+++
LOVO	+++
COLO-205	+++++
COLO-206F	++++

Invasion was analyzed using the Boyden chamber based cell invasion assay. Transmigrated cells were stained with crystal violet and counted. Different invasive capacity is indicated by the number of+symbols (>1000 cells: ++++++; >500 cells: +++++; >100 cells: ++++; >50 cells: +++; >10 cells: ++; >5 cells: +). Grey shaded = “mass” type; white = “grape-like” type.

### Gene Expression is Altered in lrECM 3D “On-top” Cultivated CRC Cells

**Figure 3:**
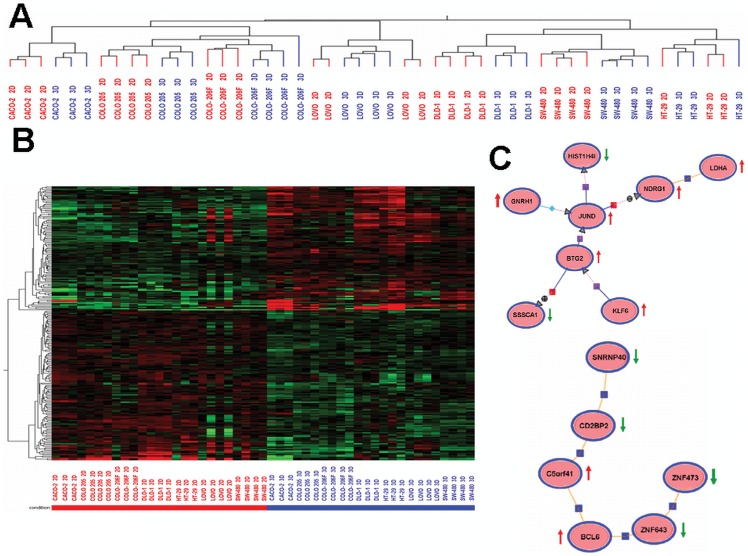
Differential gene expression in 2D and lrECM cultivated cells. **A)** Hierarchical cluster analysis of 2D and lrECM 3D on-top cultivated CRC cells lines. A total of 23000 transcripts were clustered. Each cell line builds an independent cluster for itself. With the exception for LOVO and HT-29, within each cell line two separate clusters were observed, due to the cultivation method of the cells: 2D versus lrECM 3D on-top. **B)** Heatmap of 225 significantly differentially regulated genes between 2D and lrECM 3D on-top cultivated cells. (Mann-Whitney-U test, *P*_corr_ <0.05). **C)** Literature-based network analysis of significantly differentially regulated genes (Natural Language Processor Engine, GeneSpring GX 10.5). Known interactions of 14 from a total of 225 significantly differentially regulated genes are shown. A red arrow indicates the increased expression of each gene in lrECM cultivation conditions while a green arrow indicates a reduced gene expression.

**Figure 4:**
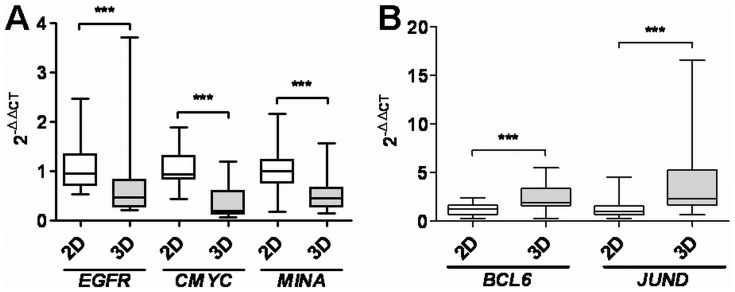
The 3D microenvironment impairs the regulation of genes involved in proliferation. Total RNA was isolated from cells cultivated in 2D and 3D microenvironments. Quantitative RT-PCR was performed and differences in gene expression levels were calculated using the 2^−ΔΔCT^ method. The mean fold change in expression of the target gene in 2D or 3D culture conditions was calculated using 2^−ΔΔCT^, where ΔΔCT = (CT _Target_ – C _GAPDH_)_culture condition_ - (CT _Target_ - C _GAPDH_)_2D_. 2^−ΔΔCT^-values of all CRC cell lines (n = 7) were pooled and are presented as a box plot. **A)** Indicates genes being downregulated in 3D microenvironments, whereas **B)** shows upregulated genes. Two-tailed *P*-values were calculated by the Mann-Whitney-U test (*** indicates a *P*-value <0.0001).

**Figure 5:**
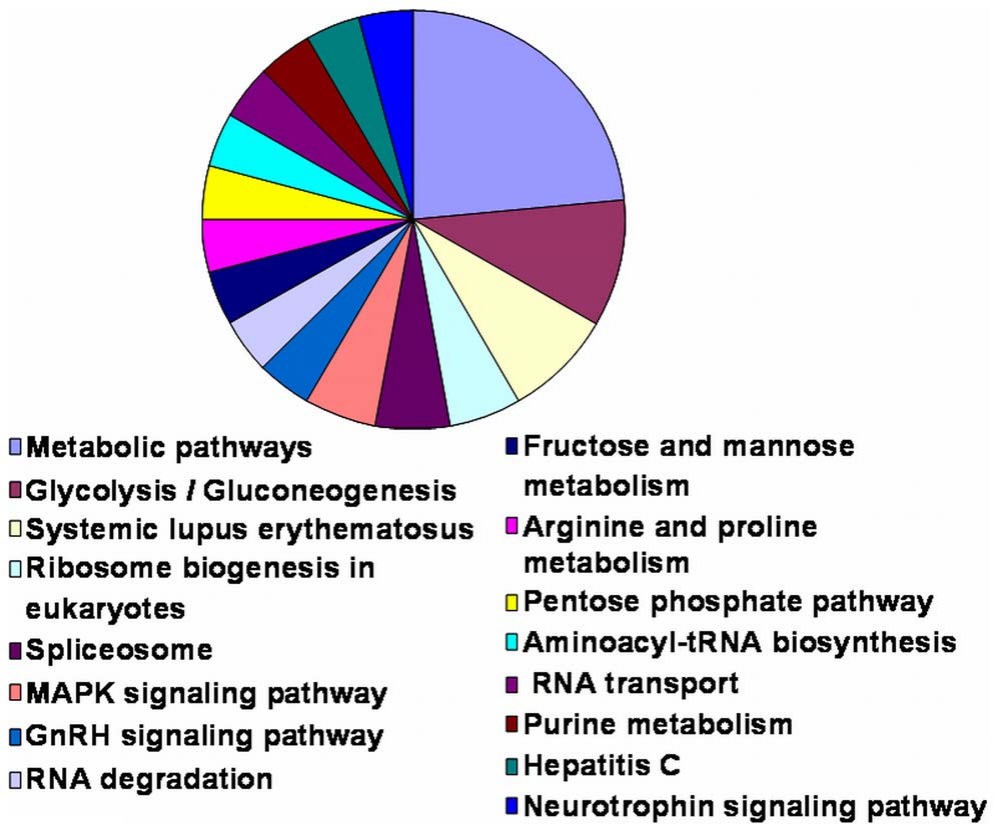
KEGG pathway analysis of genes distinguishing cells grown in 2D and lrECM culture conditions. Shown are all categories (pathways/hits) represented by three or more differently regulated genes when comparing 2D and lrECM 3D culture conditions.

The observation that CRC cell lines not only form typical spheroids in lrECM 3D but also exhibit a decreased proliferation in lrECM, suggests a general difference in gene expression levels between 2D and lrECM 3D cultures. To identify differentially expressed genes when comparing 2D and lrECM 3D cultivated cells, we investigated the transcriptome of cells growing under 2D and lrECM 3D conditions by using the Agilent Human 8×60 K High Density Oligonucleotide Microarray platform. Therefore, four independent experiments were performed in which SW-480, HT-29, DLD-1, LOVO, CACO-2, COLO-205 and COLO-206F cells were cultivated under 2D or lrECM 3D conditions, respectively. In total, 49 samples from 7 different cell lines were analyzed, 25 obtained from 2D and 24 from lrECM 3D cultures. First, we performed a hierarchical cluster analysis of cells cultured under 2D or lrECM 3D conditions. As shown in **Figure 3A**, each cell line exhibited a characteristic transcriptome profile, independent of the cell culture method. However, except for HT-29 and LOVO, within each cell line cluster, the 2D versus lrECM 3D culture conditions were clearly separated, indicating differently regulated genes between 2D and lrECM 3D cultures. Subsequent analysis revealed that 225 genes were expressed at significantly different levels when comparing cells maintained in 2D and lrECM 3D cultures **(** **Figure 3B** **)**. Pathway analysis using GeneSpring GX 10.5 software identified 14 differentially regulated genes that are known to interact with each other. Interestingly, most of these genes such as *JUND, BTG2, GNRH1, NDRG1* and *BCL6* are directly involved in signalling pathways regulating proliferation and apoptotic cell death **(** **Figure 3C** **)**. The observation that culture microenvironment impairs the regulation of genes being involved in proliferation was further confirmed by quantitative RT-PCR analysis of *EGFR*, *CMYC*, *MINA*, *JUND* and *BCL6*, detecting decreased levels of *EGFR*, *CMYC* and *MINA* **(** **Figure 4A** **)** but increased levels of *JUND* and *BCL6* **(** **Figure 4B** **)** in cells cultured on lrECM 3D substrata. Moreover, we examined the expression of Erbb3, another member of the EGFR-family as well as EGFR-ligands amphiregulin (*AREG*) and HB-EGF on mRNA levels. Interesting to us, we observed also a reduction of mRNA expression for these genes in lrECM 3D cultures (Figure S1). Since EGFR is known to be a transcriptional target of β-catenin we performed western blot analysis and detected activated (non-phosphorylated) as well as total β-catenin with specific antibodies. However, we did not detect a homogenously difference in expression levels of activated β-catenin in 2D cultured CRC cell lines when compared to cells grown under 3D conditions (Figure S2 A and B). In addition, KEGG pathway analysis of the 225 differentially regulated genes between 2D and lrECM 3D revealed that they belonged to 78 defined signalling pathways (data not shown). 16 out of these 78 pathways were represented by three or more genes and are shown in **figure 5**. The “metabolic pathway” was comprised of the most significantly regulated genes **(** **Figure 5** **)**. However, due to the noted reduced proliferation in lrECM 3D and in the context of targeted therapy in CRC, we found it most interesting that the MAPK signalling pathway was among the 16 pathways with more than three differently regulated genes.

**Figure 6:**
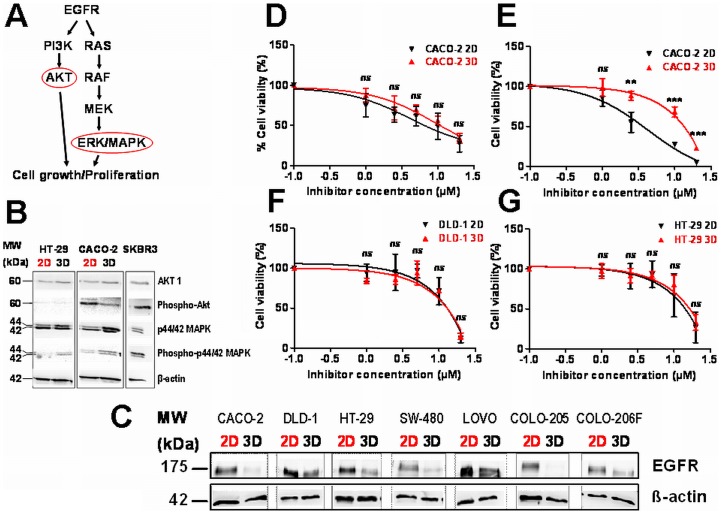
Influence of culture conditions on EGFR signaling molecules and EGFR inhibition of CRC cells. **A)** Schematic representation of the EGFR signalling pathway. **B)** Immunoblot analysis of AKT, phospho-AKT (S473), p44/42 MAPK and phospho p44/42 MAPK (Thr202/Tyr204). Equal amounts of total protein isolated from cells cultivated as 2D or lrECM 3D cultures were analyzed by SDS/PAGE/immunoblotting as indicated. β−actin served as loading control. SKBR3 served as a positive control for the expression of EGFR signaling molecules. **C)** Expression of the EGFR protein in 2D or lrECM (3D) cultivated CACO-2, DLD-1, HT-29, SW-480, LOVO, COLO 205 and COLO-206F cells. Cell lysate (40 µg protein per lane) was analyzed by SDS/PAGE/immunoblotting using monoclonal anti EGFR antibody. β-actin served as loading control. **D–G)** Treatment of 2D or lrECM cultivated CRC cells with AG1478. Two-tailed *P*-values were calculated by the paired t-test (** indicates a *P*-value <0.001; *** indicates a *P*-value <0.0001; ns = not significant). CACO-2 cells were treated for 48 h **(D)** or 96 h **(E)** with different concentrations of AG1478 as indicated. **F–G)** DLD-1 and HT-29 cells were incubated with the EGFR inhibitor AG1478 for 48 h.

Since EGF-receptor stimulates proliferation via MAP-kinases **(** **Figure 6A** **)**, which has been established as therapeutic target in the treatment of advanced CRC, we investigated the protein expression and activation patterns of EGFR and downstream activated kinases AKT and p42/44 MAPK. Accordingly, proteins were isolated from CACO-2, in which the *EGFR*, *KRAS* and *BRAF* wild type is preserved and HT-29 exhibiting only the oncogenic and activating *BRAF* mutation *V600E*. Whereas increased levels of phosphorylated AKT was detectable in CACO-2 cells growing as 2D monolayer, we observed an increase of phosphorylated p42/44 MAPK but also higher levels of total p42/44 MAPK in both cell lines grown under lrECM 3D conditions **(** **Figure 6B** **)**. SKBR3 cells served as positive control for expression levels of the distinct proteins being activated by the EGFR signalling pathway. To further evaluate and quantify the expression of phosphorylated p42/44 MAPK in 2D versus lrECM 3D, we focused on the expression levels of phosphorylated and total p42/44 MAPK in all investigated CRC cell lines. Thus, we measured the band intensity densitometrically and calculated more precisely the ratios between phosphorylated and total p42/44 MAPK or MEK1/2 **(Figure S3 A)**. Accordingly, we found in 6 out of 7 cell lines a remarkable increase of phosphorylated p42/44 MAPK in 2D cultured cell lines **(Figure S3 B)**. In addition, for MEK1/2 we observed an increase in phosphorylation in 4 cell lines cultured under 2D conditions and no changes in 2 cell lines, whereas CACO-2 cells exhibited increased phosphorylation of MEK1/2 if grown under lrECM 3D conditions when compared to 2 D cells **(Figure S3 C)**. Consistent with our observation of different proliferative activities when comparing 2D and lrECM 3D culture conditions, cell lines growing on lrECM exhibited a decrease in the expression of EGFR on protein levels **(** **Figure 6C** **)**.

### Efficacy of EGFR Inhibition is Impaired in Responsive CRC Cells Grown Under lrECM 3D Conditions

Since we observed alterations in the MAPK signaling pathway and a decreased protein expression of EGFR in lrECM 3D cultured CRC cell lines, we hypothesized that these differences may affect the sensitivity to EGFR kinase inhibition. To test our hypothesis, we treated CRC cells under 2D and 3D culture conditions with increasing concentrations of the EGFR tyrosine kinase inhibitor AG1478 for 48 or 96 hours and determined cell viability by using the MTT assay. Since CRC tumors with *KRAS* and *BRAF* wild type are expected to respond to anti-EGFR therapies, we took advantage of the human CRC cell CACO-2 exhibiting wild type *KRAS* and *BRAF*. HT-29 and DLD-1 served as negative controls being positively screened for an activating *BRAF* or *KRAS* mutation, respectively. Both, 2D and 3D cultured CACO-2 cells, were characterized by a dose dependent decrease in cell viability after treatment with AG1478 for 48 hours, but this effect was more pronounced in cells cultivated as 2D monolayer **(** **Figure 6** **D)**. Accordingly, the calculated IC50 was about 2 fold higher under lrECM 3D conditions (2D: 4.1 µM and 3D: 9.0 µM). Interestingly, this observation became more evident when CACO-2 cells were incubated for 96 hours **(** **Figure 6E** **)**. In contrast to cells cultivated in the 2D system, the dose response curve in lrECM 3D grown CACO-2 cells did not show the typical S-shaped curve for drug responding cells. As expected both, HT-29 as well as DLD-1 CRC cell lines exhibiting an activating *BRAF* or *KRAS* mutation, respectively, did not respond to the treatment with the EGFR inhibitor under 2D and lrECM 3D **(** **Figure 6F and G** **)**.

## Discussion

Although ECM provides the natural environment for benign and malignant epithelial cells, this crucial component is commonly omitted when permanent cancer cell lines are cultured and studied. However, from the pioneering work of Mina Bissell’s group and others we have learned that cultivating epithelial cells within lrECM may have dramatic effects on their phenotype and on their metabolic and cell signaling status when compared to conventional 2D culture on plastic dishes [2], [17], [20], [22]. It is therefore surprising that only relatively few studies published so far, that addressed the systematic analysis of the effects of lrECM on permanent cancer cell lines are. The few available systematic analyses focused on breast and prostate cancer. In a recent study, Kenny *et al.* analyzed systematically the effect of lrECM on several breast cancer cell lines and observed that all of them formed spheroids and therefore categorized their morphology into four distinct growth patterns [2]. The morphology ranged from benign (round morphology) appearing spheroids with well-organized nuclei to very malignant appearing formations with disorganized nuclei and invasive processes. For our systematic study of lrECM effects on CRC cell lines, we used not only this morphologic spheroid classification system, but also the same lrECM 3D ‘on-top’ assay as culture method [2], [17], [22] in order to gain comparable data, but in a different tumor entity. The collections of CRC cell lines for this study are well established and commonly used. Nevertheless, STR-analysis was performed confirming the genotype of these CRC cell lines **(Table S2)**.

In this first systematic investigation of lrECM growth effects on CRC cell lines we observed two of the four described morphological categories, the ‘mass’ type and the ‘grape-like’ type. Obviously, we cannot exclude, that the two other morphologic types can be found in other CRC cell lines not included into this study. However, the spheroid morphology of the CRC cell lines studied here was highly reproducible. This was supported by our transcriptome analysis, showing not only cell-line specific gene expression profiles, but also similar expression patterns for the lrECM 3D-cultured cells within the “cell-line clusters” in at least three independent consecutive experiments. Notably, all CRC cell lines derived from metastases displayed a ‘grape-like’ morphology upon lrECM culture, whereas all cell lines of the ‘mass’ type morphology were established from primary tumors. This correlation was statistically significant, p = 0.028, as calculated by Fishers-Exact-test. This was a striking finding, considering the fact that in the study by Kenny *et al.* on breast cancer cell lines eight of the nine ‘grape-like’ lines were derived from metastases, while five of the seven ‘mass’ types were established from primary tumors [2]. These data suggest characteristic properties retained by permanent cell lines of primary tumors and metastases that become discernable only when the cells were grown in an appropriate microenvironment, i.e. lrECM. Here, these properties of metastases-derived cells seem to lead to relatively loose cell-cell contacts [2] resulting in the typical grape-like morphology of their spheroids. In this context, it is of note that the metastases derived CRC cell lines COLO-206F and LOVO shared the ‘grape-like’ morphology upon lrECM culture, but displayed completely different morphologies when cultured under 2D conditions on plastic. Concerning proliferation, migration and invasive capacity no obvious difference was detected between primary tumor and metastases derived cell lines.

However, the lrECM 3D on-top culture method did not only reveal significant effects on the morphology of the CRC cell lines, but also on their proliferative potential and gene expression pattern. In direct comparison all CRC cell lines cultured in lrECM displayed a significantly decrease in proliferation when compared to their 2D counterparts. This might be explained by the molecular signaling from the ECM as well as from the surrounding cells, since a reduced proliferation has been reported also for colonospheres derived from CRC cell lines cultured without ECM [28]. Furthermore, cell proliferation might be reduced in CRC spheres due to enhanced differentiation status of spheres compared to 2D cultured CRC cells as well as by altered gene expression. MAPK signaling pathway, which is a downstream target of EGF receptor, was within the pathways of differently regulated genes between the two culture models. Altogether, 225 genes were significantly differentially regulated between the two cell culture models, belonging to 78 different pathways according to KEGG pathway analysis, which included the MAPK pathway. Thus, we focused on MAPK pathway because of its importance for CRC therapy. Because subsequent gene-specific analysis revealed decreased EGFR expression in lrECM cultured cells, we speculated whether this was associated with an altered proliferative response to anti-EGFR therapy. From our CRC cell line panel, we used CACO-2 cells for the EGFR-inhibition experiments because of their wild type *KRAS* and *BRAF*, respectively. Further we investigated EGFR-inhibition in DLD-1 and HT-29 cells, exhibiting mutations for either *KRAS* or *BRAF*. *KRAS* and *BRAF* mutational status of patients is usually assessed before EGFR treatment on the basis that only patients exhibiting *BRAF* and *KRAS* wild type profit from treatments with anti-EGFR therapies [29], [30]. Using the EGFR inhibitor AG1478 we observed a significantly decreased responsiveness for CACO-2 cells cultured in the lrECM on-top assay as compared to 2D cultured cells after 48 hours of treatment. If this effect can be attributed to the reduced EGFR protein expression alone and/or an interfering ECM-signaling remains speculative. Our data on EGFR inhibition in CRC are in line with a previous study testing HER-2 directed drugs in breast cancer cell lines cultured with the lrECM 3D on-top assay [31]. However, depending on the cell culture condition and the cell line a significantly decreased as well as enhanced sensitivity to treatments with anti-HER2 for 48 hours was noted. Strikingly, in this study all four investigated cell lines switched between PI3K-AKT- and RAS-MAPK-pathway activation in 2D *vs.* lrECM 3D environments [31]. The 96 hours anti-EGFR treatment assays showed that lrECM (3D) cultured CRC cells did not respond to the treatment at all. The observation that a drug response of lrECM 3D cultivated CACO-2 cells became less obvious after 96 hours might be explained by our observation that spheroid formation was more pronounced after 96 hours (data not shown) hampering drug invasion or by the elevated phospho-MAPK protein level in lrECM cultivated cells. In this context, it is again noteworthy that MAPK signaling pathway was within the pathways of differently regulated genes between 2D and lrECM 3D cultivated cells as shown by Agilent Array analysis. This rapid adaption to different environments might be an important mechanism of cancer cells to acquire therapy resistance during systemic cancer progression. As expected, neither HT-29 nor DLD-1 cells did respond to the anti-EGFR treatment for either culture condition which can be explained by their *KRAS* and *BRAF* mutational status. Interestingly, we observed not only the altered gene expression patterns in CRC cell lines growing under lrECM environment, but also altered EGFR protein expression as well as phospho-AKT and phosphor-MAPK protein levels. Thus, changes in protein expression levels and activation status add a further level of complexity to the adaption of cancer cells to different environments and may also affect the success of molecular therapies.

In summary, we present here the first systematic analysis of ECM-effects on the phenotype and genotype of commonly used CRC cell lines. To model the ECM we used the standardized lrECM 3D on-top assay [2], [17] and found, similar to previous investigations in breast cancer, significant morphologic differences between the spheroids of cell lines derived from primary tumors and metastases. The lrECM 3D environment resulted in a dramatically altered phenotype and significant changes in gene expression patterns that was in line with the sensitivity to anti-EGFR targeted therapy. The presented study underscores the importance to model the ECM microenvironment and provides a basis to work with more realistic CRC *in vitro* models.

## Supporting Information

Figure S1 **The 3D microenvironment impairs the regulation of EGF-family members.** Total RNA was isolated from cells cultivated in 2D and 3D microenvironments. Quantitative RT-PCR was performed and differences in gene expression levels were calculated using the 2^−ΔΔCT^ method. The mean fold change in expression of the target gene in 2D or 3D culture conditions was calculated using 2^−ΔΔCT^, where ΔΔCT = (CT _Target_ – C _GAPDH_)_culture condition_ − (CT _Target_ − C _GAPDH_)_2D_. 2^−ΔΔCT^-values of all CRC cell lines (n = 7) were pooled and are presented as box plot. Two-tailed *P*-values were calculated by the Mann-Whitney-U test (*** indicates a *P*-value <0.0001).
(TIF)

Figure S2 **Influence of culture conditions the activity of β-catenin. A)** Immunoblot analysis of non-phosphorylated (active β-catenin) and total β-catenin. Equal amounts of total protein isolated from cells cultivated as 2D or lrECM 3D cultures were analyzed by SDS/PAGE/immunoblotting as indicated. **B)** Ratios between active β-catenin and total β-catenin were calculated by using the densitometric measured band intensity. White bars represent cells cultured on on lrECM (3D), black bars cells cultured plastic (2D).
(TIF)

Figure S3 **Influence of culture conditions on the MAPK signaling pathway. A)** Immunoblot analysis of p44/42 MAPK, phospho p44/42 MAPK (Thr202/Tyr204), MEK1/2 and phospho MEK1/2 (Ser217/221). Equal amounts of total protein isolated from cells cultivated as 2D or lrECM 3D cultures were analyzed by SDS/PAGE/immunoblotting as indicated. Ratios between phosphorylated and total protein for **B)** p42/44 MAPK and **C)** MEK1/2 were calculated by using the densitometric measured band intensity. White bars represent cells cultured on on lrECM (3D), black bars cells cultured plastic (2D).
(TIF)

Table S1 Primers and probes used for quantitative RT-PCR.
(DOC)

Table S2 **STR Analysis of investigated cell lines.** STR Analysis revealed that the genotype of the cell line was identical to the genotype of the presumed parental cells. The genotypes were compared to genotypes outlined by the different cell culture centers. The deviation (*) from the reported genotype in CACO-2 cells might be explained by Y-chromosome loss in a subclone due to genetic instability.
(DOC)
