# Supplementary figures and images for: Impact of the 3D Microenvironment on Phenotype, Gene Expression, and EGFR Inhibition of Colorectal Cancer Cell Lines
Source: PLoS One. 2013 Mar 26;8(3):e59689. doi: 10.1371/journal.pone.0059689 (PMC3608563; doi:10.1371/journal.pone.0059689)

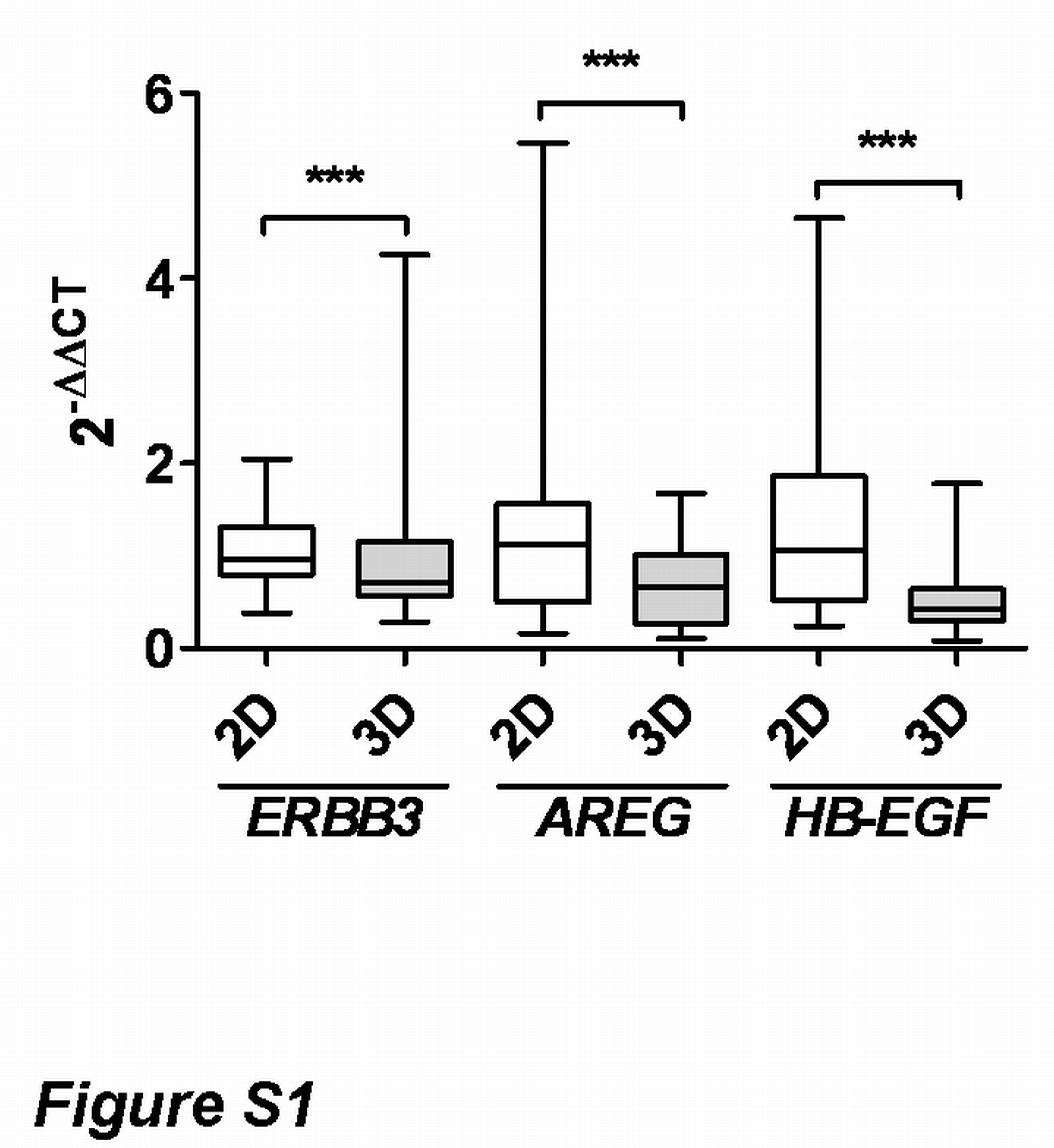

Supplement: Figure S1 — The 3D microenvironment impairs the regulation of EGF-family members. Total RNA was isolated from cells cultivated in 2D and 3D microenvironments. Quantitative RT-PCR was performed and differences in gene expression levels were calculated using the 2−ΔΔCT method. The mean fold change in expression of the target gene in 2D or 3D culture conditions was calculated using 2−ΔΔCT, where ΔΔCT = (CT Target – C GAPDH)culture condition − (CT Target − C GAPDH)2D. 2−ΔΔCT-values of all CRC cell lines (n = 7) were pooled and are presented as box plot. Two-tailed P-values were calculated by the Mann-Whitney-U test (*** indicates a P-value <0.0001). (TIF) [file pone.0059689.s001.tif]

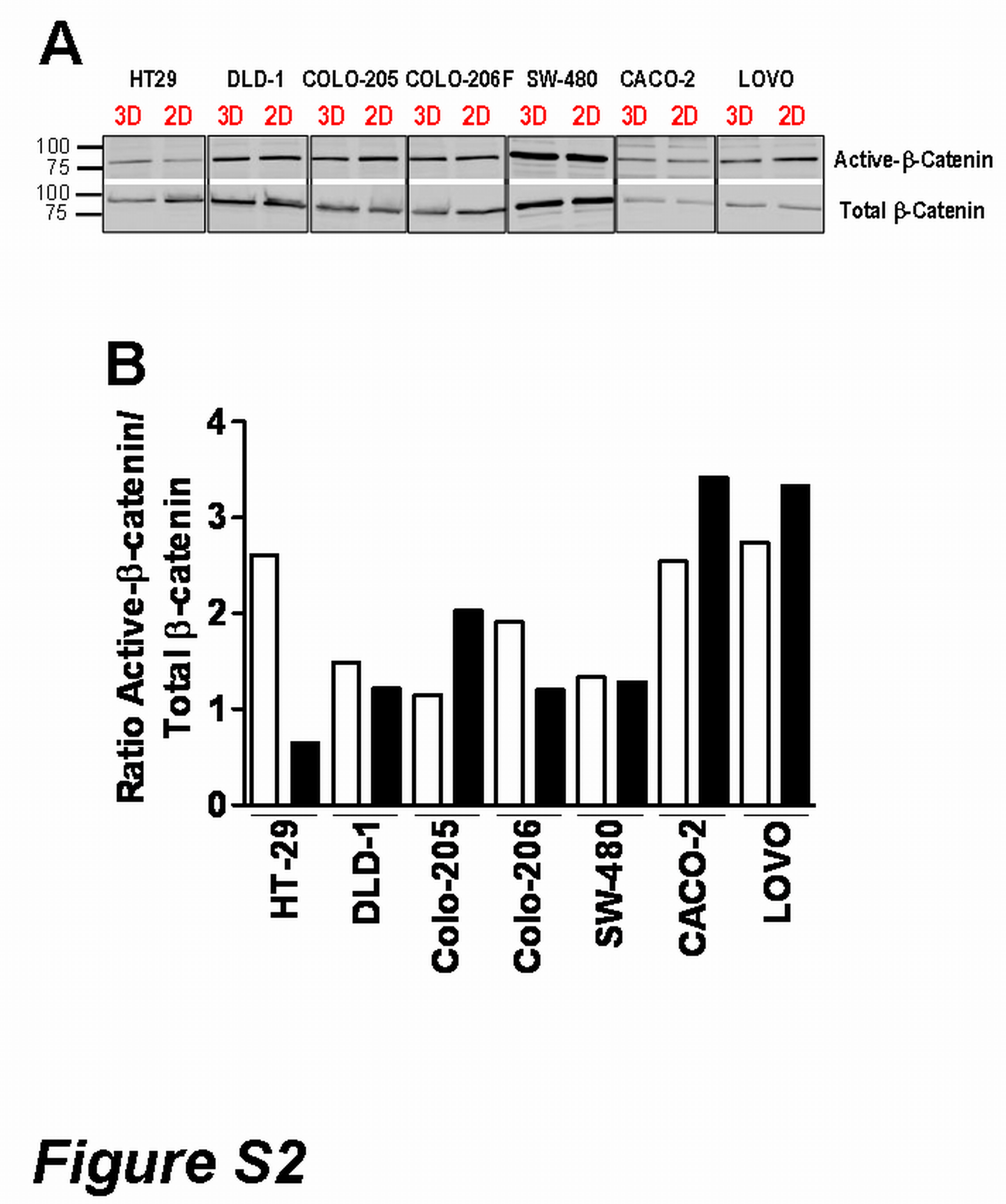

Supplement: Figure S2 — Influence of culture conditions the activity of β-catenin. A) Immunoblot analysis of non-phosphorylated (active β-catenin) and total β-catenin. Equal amounts of total protein isolated from cells cultivated as 2D or lrECM 3D cultures were analyzed by SDS/PAGE/immunoblotting as indicated. B) Ratios between active β-catenin and total β-catenin were calculated by using the densitometric measured band intensity. White bars represent cells cultured on on lrECM (3D), black bars cells cultured plastic (2D). (TIF) [file pone.0059689.s002.tif]

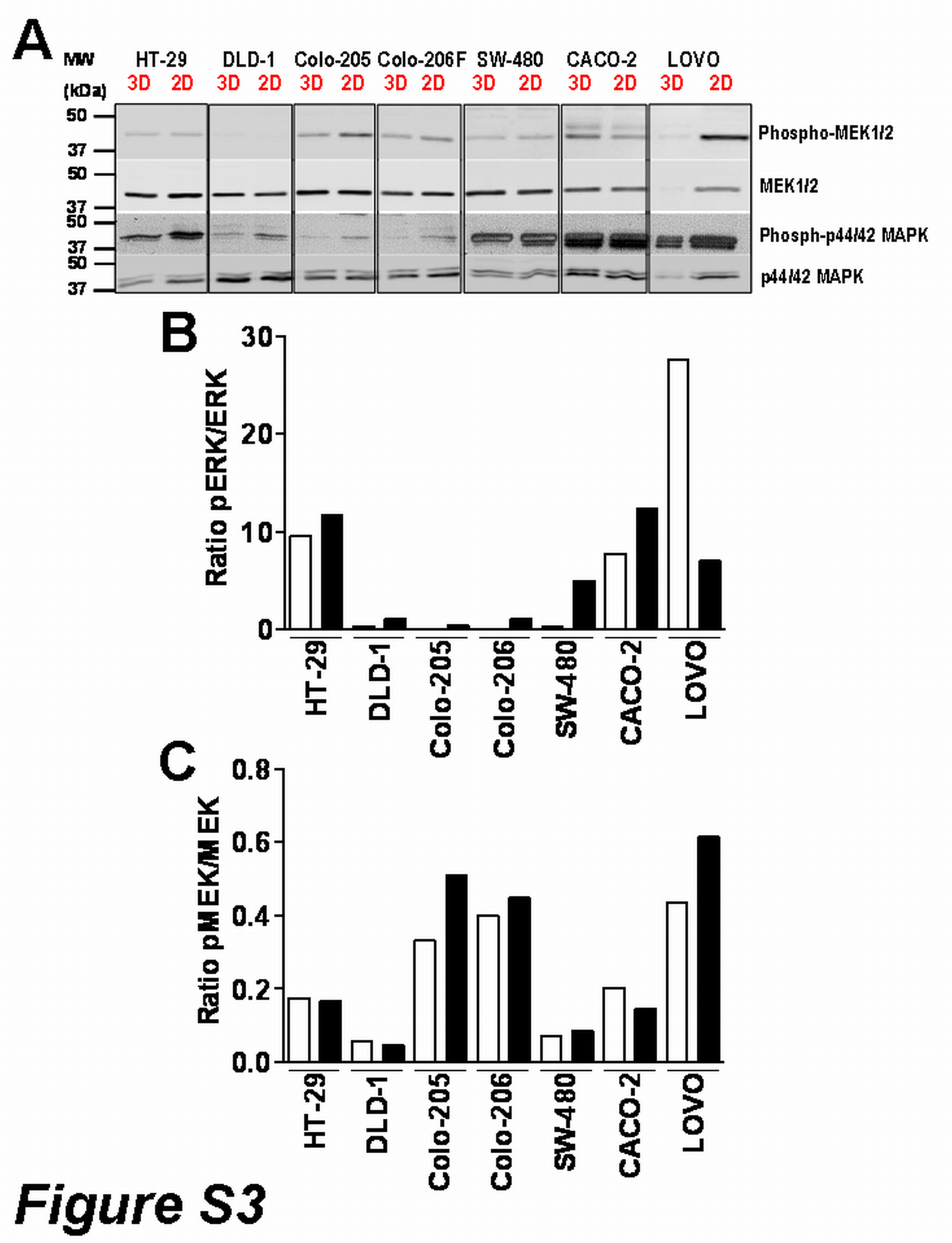

Supplement: Figure S3 — Influence of culture conditions on the MAPK signaling pathway. A) Immunoblot analysis of p44/42 MAPK, phospho p44/42 MAPK (Thr202/Tyr204), MEK1/2 and phospho MEK1/2 (Ser217/221). Equal amounts of total protein isolated from cells cultivated as 2D or lrECM 3D cultures were analyzed by SDS/PAGE/immunoblotting as indicated. Ratios between phosphorylated and total protein for B) p42/44 MAPK and C) MEK1/2 were calculated by using the densitometric measured band intensity. White bars represent cells cultured on on lrECM (3D), black bars cells cultured plastic (2D). (TIF) [file pone.0059689.s003.tif]
