# Supplementary material for: Impact of the 3D Microenvironment on Phenotype, Gene Expression, and EGFR Inhibition of Colorectal Cancer Cell Lines
Source: PLoS One. 2013 Mar 26;8(3):e59689. doi: 10.1371/journal.pone.0059689 (PMC3608563; doi:10.1371/journal.pone.0059689)
Supplement: Table S1 — Primers and probes used for quantitative RT-PCR. (DOC) [file pone.0059689.s004.doc]

**Table S1: Primers and probes used for quantitative RT-PCR**

| **Primer** | **5`-3`Sequence** | **Universal Probe Roche** |
| --- | --- | --- |
| GAPDH forward | AGCCACATCGCTCAGAC | 60 |
| GAPDH reverse | GCCCAATACGACCAAATC | 60 |
| BCL6 forward | CCG GAA GTT TAT TAA GGC CAG T | 87 |
| BCL6 reverse | CCC GAT AGG CCA TGA TGT | 87 |
| EGFR forward | ACA CAG AAT CTA TAC CCA CCA GAG T | 50 |
| EGFR reverse | ATC AAC TCC CAA ACG GTC AC | 50 |
| CMYC forward | GCT GCT TAG ACG CTG GAT TT | 66 |
| CMYC reverse | TAA CGT TGA GGG GCA TCG | 66 |
| JUND forward | CAG CGA GGA GCA GGA GTT | 81 |
| JUND reverse | GAG CTG GTT CTG CTT GTG TAA AT | 81 |
| MINA forward | CCC CAT TAT GAT GAT GTC GAG | 78 |
| MINA reverse | GAG GCG CCA GTG TTT CTC T | 78 |
| AREG forward | tga tcc tca cag ctg ttg ct | 73 |
| AREG reverse | tcc att ctc ttg tcg aag ttt ct | 73 |
| ERB3 forward | ctg atc acc ggc ctc aat | 37 |
| ERB3 reverse | gga aga cat tga gct tct ctg g | 37 |
| HB-EGF forward | tgg ggc ttc tca tgt tta gg | 55 |
| HB-EGF reverse | cat gcc caa ctt cac ttt ctc | 55 |
