# Supplementary material for: Impact of the 3D Microenvironment on Phenotype, Gene Expression, and EGFR Inhibition of Colorectal Cancer Cell Lines
Source: PLoS One. 2013 Mar 26;8(3):e59689. doi: 10.1371/journal.pone.0059689 (PMC3608563; doi:10.1371/journal.pone.0059689)
Supplement: Table S2 — STR Analysis of investigated cell lines. STR Analysis revealed that the genotype of the cell line was identical to the genotype of the presumed parental cells. The genotypes were compared to genotypes outlined by the different cell culture centers. The deviation (*) from the reported genotype in CACO-2 cells might be explained by Y-chromosome loss in a subclone due to genetic instability. (DOC) [file pone.0059689.s005.doc]

**Table S2: STR Analysis of investigated cell lines.** STR Analysis revealed that the genotype of the cell line was identical to the genotype of the presumed parental cells. The genotypes were compared to genotypes outlined by the different cell culture centers. The deviation (*) from the reported genotype in CACO-2 cells might be explained by Y-chromosome loss in a subclone due to genetic instability.

| **STR analysis** | **CACO-2** | **COLO-205** | **COLO-206F** | **DLD-1** | **HT-29** | **LOVO** | **SW-480** |
| --- | --- | --- | --- | --- | --- | --- | --- |
| MY | X* | X | X | XY | XX | XY | X |
| VWA | 16-18 | 15 | 15 | 18-19 | 17-19 | 17-18 | 16 |
| SE33 | 21 | 18 | 18 | 18-27.2 | 21 | 19.2-25.2 | 19.2-30.2 |
| TH01 | 6 | 8-9 | 8-9 | 7-9.3 | 6-9 | 9.3 | 8 |
| D21S11 | 30 | 30.2-33.2 | 30.2-33.2 | 29-32.2 | 29-30 | 29-31.2 | 30-30.2 |
| D8S1179 | 12 | 9-14 | 9-14 | 15 | 10-16 | 10 | 13 |
| D3S1358 | 14 | 16 | 16 | 17 | 15-17 | 14-17 | 15 |
| FGA | 19 | 21-23 | 23 | 22 | 20-22 | 18-20 | 24 |
| D18S51 | 12 | 18 | 18 | 11-17 | 13 | 13-18 | 13 |
| D19S433 | 15 | 13-14 | 13-14 | 14-16 | 14 | 14-15 | 13 |
| TPOX | 9-11 | 11 | 11 | 8-11 | 8-9 | 8 | 11 |
| D16S539 | 12-13 | 12-13 | 12-13 | 12-13 | 11-12 | 9-12 | 13 |
| D5S818 | 12 | 10-13 | 10-13 | 13 | 11-12 | 11-13 | 13 |
| D2S1338 | 30 | 17-18 | 17-18 | 17-25 | 19-23 | 17-18 | 17-24 |
| Ordered from | DSMZ | ATCC | DSMZ | DSMZ | DSMZ | ECACC | DSMZ |
